# Deep medullary vein abnormalities associated with cognitive function and Alzheimer's disease plasma biomarkers in dementia‐free older adults: The MIND‐China Study

**DOI:** 10.1002/alz70860_102602

**Published:** 2025-12-23

**Authors:** Dejia Yin, Jiafeng Wang, Chunyan Li, Xiaoyu Wang, Yan Wang, Qianqian Xie, Huisi Zhang, Ziwei Chen, yongxiang wang, Tingting Hou, Lin Cong, Li Zhou, Yifeng Du, Lin Song, Chengxuan Qiu

**Affiliations:** ^1^ Weifang People's Hospital, Shandong Second Medical University, Weifang, Shandong, Weifang, Shandong, China; ^2^ Shandong Provincial Hospital Affiliated to Shandong First Medical University, Jinan, Shandong, China; ^3^ Shandong Provincial Clinical Research Center for Geriatric Neurological Diseases, Jinan, Shandong, China; ^4^ Key Laboratory of Endocrine Glucose & Lipids Metabolism and Brain Aging, Ministry of Education; Department of Neurology, Shandong Provincial Hospital Affiliated to Shandong First Medical University, Jinan, Shandong, China; ^5^ Shandong Provincial Clinical Research Center for Neurological Diseases, Jinan, Shandong, China; ^6^ Medical Science and Technology Innovation Center, Shandong First Medical University & Shandong Academy of Medical Sciences, Jinan, Shandong, China; ^7^ Shandong Provincial Hospital affiliated to Shandong First Medical University, Jinan, Shandong, China; ^8^ Institute of Brain Science and Brain‐Inspired Research, Shandong First Medical University & Shandong Academy of Medical Sciences, Jinan, Shandong, China; ^9^ Aging Research Center, Department of Neurobiology, Care Sciences and Society, Karolinska Institute‐Stockholm University, Solna, Sweden; ^10^ Department of Neurology, Shandong Provincial Hospital Affiliated to Shandong First Medical University, Jinan, Shandong, China; ^11^ Department of Neurology, Shandong Provincial Hospital affiliated to Shandong First Medical University, Jinan, Shandong, China; ^12^ Aging Research Center, Karolinska Institutet and Stockholm University, Stockholm, Sweden; ^13^ Aging Research Center and Center for Alzheimer Research, Karolinska Institutet‐Stockholm University, Stockholm, Sweden

## Abstract

**Background:**

The deep medullary veins (DMVs) play a pivotal role in maintaining normal perfusion of white matter, and thus, discontinuation of DMVs may be involved in the pathogenesis of cerebral small vessel disease. Evidence has emerged that abnormalities of DMVs are associated with cognitive impairment. We sought to investigate the associations of DMV score with multi‐dimensional cognitive outcomes and blood biomarkers for brain aging among older adults.

**Method:**

This population‐based cross‐sectional study used data from 1206 dementia‐free participants (age≥60 years) in MIND‐China MRI substudy; of these, plasma amyloid‐β, total tau, and neurofilament light chain were measured in 903 individuals using the SIMOA platform. The DMVs were evaluated using the SWI sequence. We assessed six segments that included the bilateral frontal lobes, parietal lobes, and occipital lobes. The total DMV score was calculated as the sum of the DMV scores from all six segments. A higher score indicated low DMV visibility. We used a neuropsychological test battery to assess cognitive function. The Petersen's criteria were used to define mild cognitive impairment (MCI). Data were analyzed using logistic regression, linear regression, and mediation models.

**Result:**

A higher DMV score was significantly associated with increased likelihoods of MCI (OR=1.19; 95%CI=1.06‒1.34) and amnestic mild cognitive impairment (aMCI) (1.21; 1.06‒1.37), and lower scores in MMSE (‐0.01; ‐0.02‒0.00; *p* = 0.041), global cognition (‐0.05; ‐0.08‒‐0.03), memory (‐0.06; ‐0.10‒‐0.02), verbal fluency (‐0.05; ‐0.08‒‐0.02), attention (‐0.04; ‐0.07‒‐0.01), and executive function (‐0.06; ‐0.10‒‐0.03). In the plasma biomarker subsample, a higher DMV score was significantly related to lower amyloid‐β42/amyloid‐β40 ratio (β = ‐0.11; ‐0.20‒‐0.02).

**Conclusion:**

This study suggests that the discontinuation of DMVs is associated with MCI and aMCI, poor function in global cognition and multiple cognitive domains, and Alzheimer pathology in rural Chinese older adults.

